# Autophagy defects and related genetic variations in renal cell carcinoma with eosinophilic cytoplasmic inclusions

**DOI:** 10.1038/s41598-018-28369-y

**Published:** 2018-07-02

**Authors:** Zhou Yu, Jing Ma, Xia Li, Yixiong Liu, Mingyang Li, Lu Wang, Ming Zhao, Huiying He, Yifen Zhang, Qiu Rao, Danhui Zhao, Yingmei Wang, Linni Fan, Peifeng Li, Yang Liu, Fang Liu, Feng Zhang, Jing Ye, Qingguo Yan, Shuangping Guo, Zhe Wang

**Affiliations:** 1 State Key Laboratory of Cancer Biology, Department of Pathology, Xijing Hospital and School of Basic Medicine, Fourth Military Medical University, Xi’an, 710032 China; 2 Department of Plastic and Reconstructive Surgery, Xijing Hospital, Fourth Military Medical University, Xi’an, 710032 China; 3 Department of Pathology, Zhejiang Provincial People’s Hospital, Hangzhou, 310034 China; 4 Department of Pathology, Peking University Third Hospital, Peking, 100191 China; 5 Department of Pathology, Nanjing Drum Tower Hospital, Nanjing, 210008 China; 6 Department of Pathology, Nanjing General Hospital, Nanjing, 210002 China

## Abstract

The relationship between autophagy and tumour is well studied, but tumour cell morphological changes associated with autophagy defects are rarely reported, especially in renal cell carcinoma (RCC). We collected 10 renal tumour samples with characteristic eosinophilic cytoplasmic inclusions (ECIs) and found that the ECIs were majorly composed of sequestosome 1/P62, neighbor of BRCA1 gene 1 (NBR1), PEX14, and CATALASE1 (CAT1). Further, transmission electron microscopy analysis revealed that ECIs were aggregates of proteinaceous material and peroxisomes. These results confirmed that ECIs in RCCs were the products of autophagy defects. The presence of ECIs was correlated with high Fuhrman grade components of RCCs. Whole-exome sequencing (WES) and Sanger sequencing confirmed that tumours with ECIs showed somatic mutations or high frequency of genetic variations in autophagy-related (ATG) genes, such as *ATG7*, *ATG5*, and *ATG10*. These results indicate that nucleotide changes in ATG genes are associated with autophagy defect, ECI formation, and even tumour grade in RCCs.

## Introduction

Renal cell carcinoma (RCC) accounts for 2–3% of all malignant diseases in adults^1^. However, RCCs with eosinophilic cytoplasmic inclusions (ECIs) are rarely reported^2–8^. ECIs observed in RCCs are round to oval homogeneous cytoplasmic eosinophilic globules with a clear surrounding halo. Ultrastructural examination has shown that ECIs consist of fibrillar substances surrounded by membrane-bound electron-dense organelles^7^. In other words, ECIs are a mixture of protein aggregates and/or organelles. However, the exact components of ECIs and the nature of the electron-dense organelles are still unknown.

Macroautophagy (hereafter referred to as autophagy) is an evolutionarily conserved process that mainly plays a role in damaged organelle degradation and intracellular content digestion^9^. Autophagy has been implicated in different aspects of cancer, such as tumour cell motility, invasion, cancer metastasis, epithelial-mesenchymal transition, tumour cell dormancy, maintenance of cancer stem cell phenotype, drug resistance, and immune surveillance^10–17^. However, the role of autophagy-related (ATG) gene mutations in cancers is not so well studied^17^, and the mutation profile of ATG genes associated with RCC, in particular, needs further investigation^18^. In addition, it is known that blockage of autophagy can result in the accumulation of P62 and dysfunctional organelles, such as peroxisomes and mitochondria, and microscopically visible inclusion formation^19–23^. And increased expression of autophagy-related gene 5 (ATG5), microtubule-associated protein 1 light chain 3 alpha (LC3A), microtubule-associated protein 1 light chain 3 beta (LC3B) and beclin 1 (BECN1) was observed in sporadic inclusion body myositis^24^. But despite this, the relationship between microscopically visible ECIs and abnormal autophagy has not been studied so far. Based on these gaps in the literature, the present study set out to investigate the characteristic morphological features of renal cancer related to autophagy defects and the relationship between genetic variations of ATG genes and tumour cell morphological changes in RCCs.

We investigated 10 rare cases of RCCs with characteristic ECIs and found that the ATG proteins sequestosome 1/P62, neighbor of BRCA1 gene 1 (NBR1), BECN1, ATG5 and LC3 are located in ECIs, and that peroxisomes are distributed within ECIs too. In addition, ECIs were only associated with tumour components of high Fuhrman grade. Finally, we performed whole-exome sequencing (WES) and Sanger sequencing and revealed numerous genetic variations in the ATG genes in RCCs with ECIs. And no somatic mutations were detected in ATG genes in 103 RCCs without ECIs. The results indicate that autophagy dysfunction in RCCs is related to ECI pathogenesis and tumour grade.

## Results

### Clinicopathological features of RCC with ECIs

The clinicopathological features of the 10 RCCs (including 8 clear cell RCCs [ccRCCs], 1 mucinous tubular and spindle cell carcinoma [MTSCC], and 1 papillary RCC [PRCC]) included in this study are summarized in Table 1. All the cases were of primary RCC, for which the resected kidney samples were obtained. The patients included five men and five women, who had no family history of kidney cancer. Their ages ranged from 36 to 68 years (mean = 56 years). All the patients underwent radical nephrectomy. There was no predilection for laterality. Tumour size ranged from 3 to 15 cm (mean = 6.8 cm). The pathologic TNM tumour categories were pT1b (3/10, 30%), pT2a (2/10, 20%), and pT3 (1/10, 10%). One patient had a lymph node metastasis, and 2 patients had bone metastases. Tumour grade according to the Fuhrman system was 3 in five cases (50%) and 4 in remaining five cases (50%). Fluorescence *in situ* hybridization (FISH) results indicated that two of the ten tumours displayed deletion of chromosome 3p (patient 1 and 7), and two tumours revealed trisomy of chromosome 7 or 17 (patient 9 and 10, shown in Supplementary Fig. 2). Follow-up data were available for 4 of the 10 patients. Two of the four patients were alive without disease and one was alive with disease after a follow-up period of 18 to 31 months. One patient died of the disease after 11 months.

**Table 1:** Clinicopathological features of RCC with ECIs.

Patients	Age (y)	Gender	Tumour #	Size (cm)	Side	Grade	Stage	Procedure	3p FISH	Trisomy 7 or 17	Follow-u p (mo)	Overall survival	Tumour subtype
1	62	F	1	3	R	4	NA	RN	Deletion	NA	NA	NA	ccRCC
2	48	M	1	9	R	3	T2aNX	RN	NA	NA	NA	NA	ccRCC
3	68	M	2	5	L	3	T1bNX	RN	NA	NA	NA	NA	ccRCC
4	60	M	1	8	R	4	T2aN0M1	RN	NA	NA	NA	NA	ccRCC
5	55	F	1	6.5	R	4	NA	RN	NA	NA	NA	NA	ccRCC
6	36	F	1	3.5	L	4	T1b	RN	NO	NO	31	AOD	ccRCC
7	52	M	1	5.5	R	3	T1bN0	RN	Deletion	NA	30	AWD	ccRCC
8	66	F	1	5.3	L	3	NA	RN	NA	NA	18	AOD	ccRCC
9	64	F	1	15	L	3	NA	RN	NA	YES	NA	NA	PRCC
10	53	M	1	7	L	4	T3N1M1	RN	NA	YES	11	DOD	MTSCC

Abbreviations: F, female; M, male; L, left; R, right; NA, not available; RN, radical nephrectomy; ccRCC, clear cell renal cell carcinoma; PRCC, papillary renal cell carcinoma; MTSCC, mucinous tubular and spindle cell carcinoma; DOD, dead of disease; AOD, alive without disease; AWD, alive with disease.

### Light microscopic characteristics of ECIs

**Figure 1:**
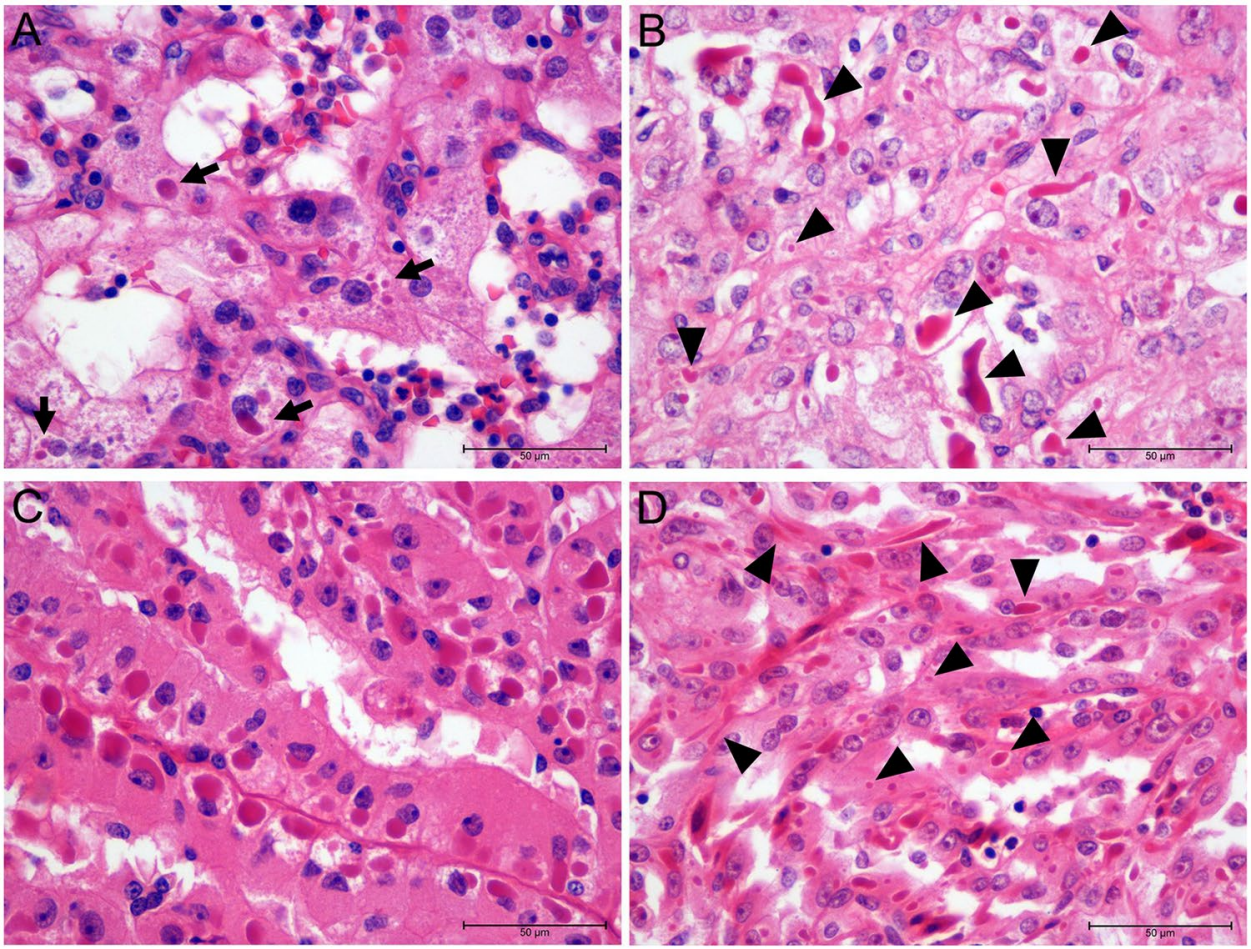
Light microscopic characteristics of ECIs in three types of RCCs (H&E, ×400). (**A** and **B**) ECIs in ccRCC. Note the variations in the number of ECIs (arrows) and their size (arrowheads) and shape (arrowheads). (**C**) Round-to-oval ECIs were observed to be regularly arranged in a juxtanuclear position in PRCC cells. (**D**) ECIs of varying sizes and shapes (arrowheads) were found in MTSCC cells.

Based on their morphological features, most of the ECIs observed in ccRCC (Fig. 1A,B), PRCC (Fig. 1C) and MTSCC (Fig. 1D) were similar in appearance. Generally, ECIs found in ccRCC (Fig. 1A,B) and MTSCC (Fig. 1D) were eosinophilic and appeared as well-circumscribed intracytoplasmic globules with a clear surrounding halo. However, their shapes were diverse and ranged from round and oval to irregular, and in the case of ccRCC, their size varied between 1 and 33 μm (greatest diameter) (arrowheads, Fig. 1B). In contrast, the ECIs in the PRCC sample were more uniform in appearance and number (Fig. 1C). The ECIs were juxtanuclear in the case of PRCC (Fig. 1C), while they were randomly distributed in the cytoplasm in the case of ccRCC and MTSCC (Fig. 1A,B and D). ECIs only existed in tumour components with high Fuhrman grade (grade 3 or 4), no ECIs were found in the matched normal tissues or tumour components with low Fuhrman grade.

### Ultrastructural features of ECIs

**Figure 2:**
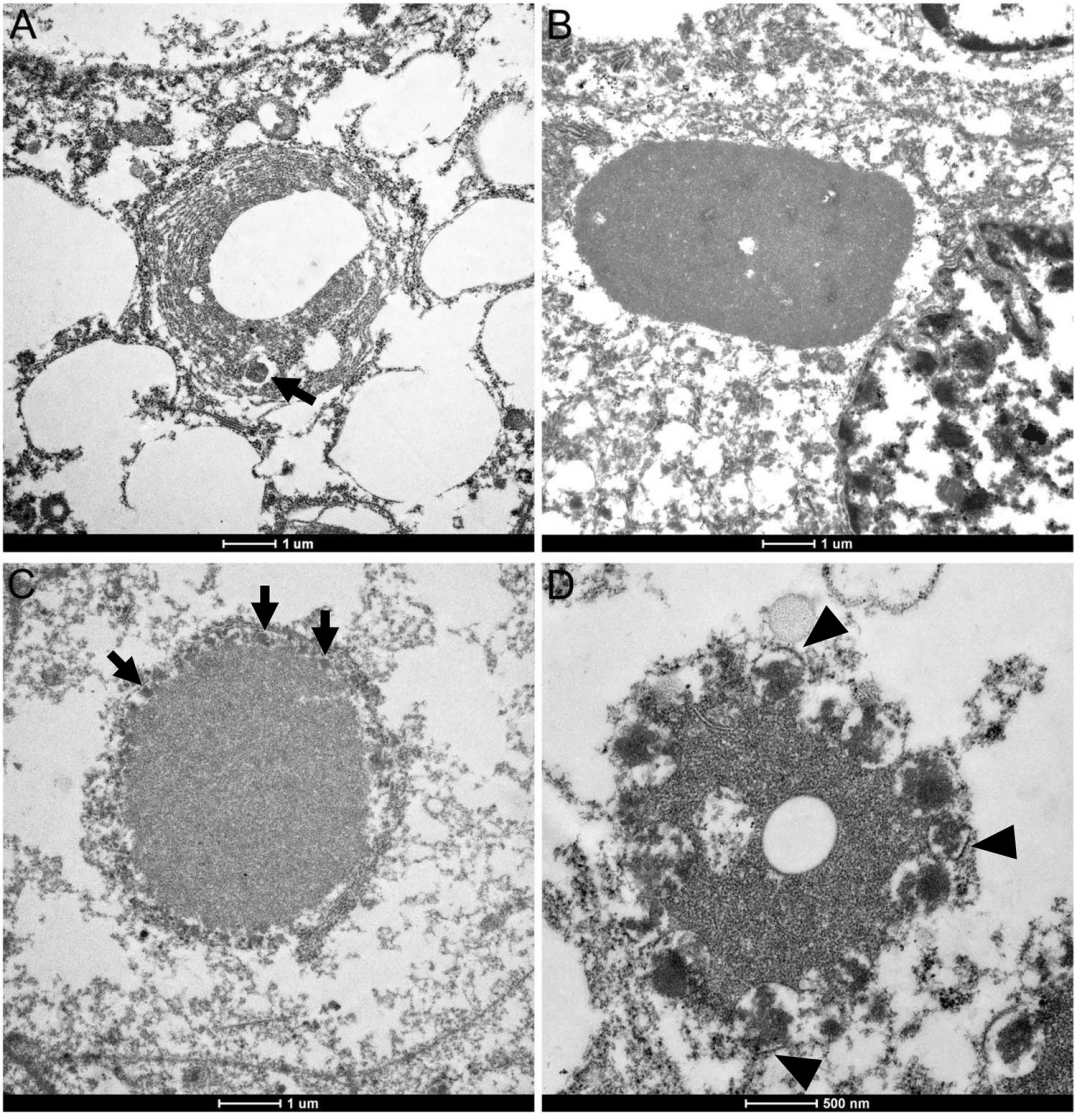
Transmission electron micrographs of ECIs in RCCs. (**A**) An ECI exhibiting a concentric membranous structure with lipid or clear spaces; note the round electron-dense structure within the concentric membranous structure (arrow). (**B**) An ECI consisting of proteinaceous material with several clear spaces within it. (**C**) An ECI comprising proteinaceous material with electron-dense structures (arrows) completely enclosing it. (**D**) An ECI displaying aggregates of fibrillar components and single membrane-bound electron-dense structures (arrowheads).

ECIs in RCC cells can easily be detected even in paraffin-embedded tissues, and a large amount of lipids and glycogen particles are also present in the cytoplasm. Some of the ECIs exhibited concentric membranous structures with central lipid droplets or some clear spaces, and a round electron-dense structure present among the concentric membranous layers could also be seen (patient 7, Fig. 2A). Some of the ECIs exhibited distinct features from the others: they were composed of homogenous proteinaceous or fibrillar materials with or without round electron-dense structures partially or completely surrounded them (Fig. 2B,C and D). ECIs without electron-dense structures were found in two cases (patient 4 and 5), while ECIs with electron-dense structures were observed in 4 cases (patient 6, 7, 8 and 9). The electron-dense structures were observed to be single membrane-bound organelles (arrowheads, Fig. 2D), which is consistent with the structure of peroxisomes. Moreover, their sizes were similar to those of peroxisomes, so we concluded that the single membrane-bound structures were peroxisomes. To confirm this deduction, further immunostaining was performed.

### ATG proteins located in ECIs

**Figure 3:**
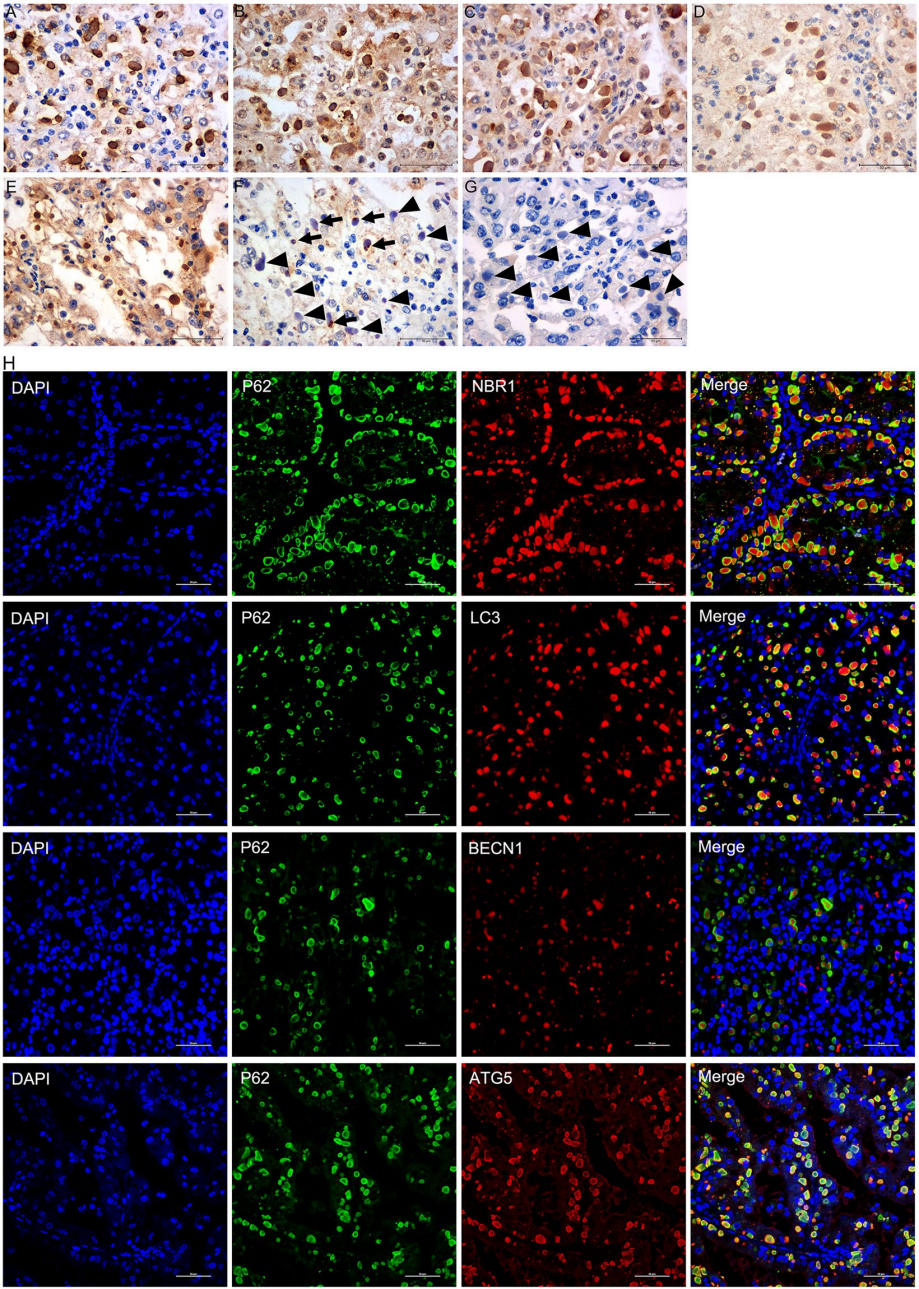
Immunohistochemical staining and double-immunofluorescence labeled microscopic images of ECIs in RCCs. Immunohistochemical staining showed that the ECIs were immunopositive for P62 (**A**), NBR1 (**B**), LC3 (**C**), BECN1 (**D**), and ATG5 (**E**), and immunonegative for LCK (arrows in **G**). Ub1 immunopositivity was detected in a small number of ECIs (arrows in **F**). Double-immunofluorescence staining of ECIs demonstrated co-localization of P62 with NBR1, LC3, BECN1 or ATG5 in ECIs (**H**).

The results of immunostaining for ATG proteins in ECIs are presented in Table 2. The cytoplasm of RCC cells showed moderate staining for P62, NBR1, LC3 and ATG5 (Fig. 3A,B,C and E) and weak staining for BECN1 and Ubiquitin1 (Ub1) (Fig. 3D and F). There was no detectable immunoreactivity for low-molecular-weight cytokeratin (LCK) in both the cytoplasm and inclusions (arrows in Fig. 3G). With regard to the ECIs, the majority were intensely immunopositive for P62; the staining pattern for BECN1 was similar, except that the stains were of moderate intensity (Fig. 3A and D). ECIs positive for NBR1, LC3 and ATG5 were detected in 80% (8/10), 67% (6/9) and 78% (7/9) of the affected cases, respectively (Table 2). The proportion of labelled ECIs was variable in each case, and the highest labelling efficiency was observed for NBR1. Moreover, P62 immunopositivity was mainly observed at the borders of ECIs (Fig. 3A), while almost homogenous staining for NBR1, LC3, BECN1 and ATG5 was found in all the inclusions. Antigen expression of Ub1 was only detected in a minor proportion of ECIs in four cases (Table 2 and Fig. 3F [arrows]) with weak or moderate staining intensity, while the majority did not exhibit Ub1 expression (Table 2 and Fig. 3F [arrowheads]). Double-immunofluorescence labeling showed co-localization of P62 with NBR1, LC3, BECN1 or ATG5 in a large number of ECIs (Fig. 3H). These results demonstrate that P62, BECN1, NBR1, LC3 and ATG5 are components of ECIs. However, with the exception of P62, the rest of the proteins may not be indispensable for ECI formation, since some of the ECIs were not positive for these proteins.

**Table 2:** Results of ECI immunostaining.

Patient	P62	NBR1	UB1	LCK	LC3	BECN1	ATG5	LAMP1	LAMP2	PEX14	CAT1	TOM20	CALR	RPS6	GM130	RAB5A
1	++++	+++	NA	NA	NA	NA	NA	NA	NA	NA	+++	NA	NA	NA	NA	NA
2	++++	+++	+	−	−	+++	+++	−	−	+++	+++	−	−	−	−	−
3	++++	++++	++	−	++	++++	++++	−	−	++++	++++	−	−	−	−	−
4	++++	−	−	−	−	++++	+++	−	−	−	−	−	−	−	−	−
5	++++	−	−	−	+	++++	+++	−	−	−	−	−	−	−	−	−
6	++++	++++	−	−	+++	++++	++	−	−	++++	+++	−	−	−	+++	−
7	++++	++++	+	−	−	++++	++++	−	−	++++	++++	−	−	−	++	−
8	++++	++++	+	−	+++	++++	−	−	−	++++	+++	−	−	−	+	−
9	++++	+++	−	−	+++	+++	+++	−	−	+++	++	−	−	−	++	−
10	++++	++++	−	−	++	++++	−	−	−	++++	+++	−	−	−	−	−

Notes: −, all ECIs were negative; +, 1–25% ECIs were positive; ++, 26–50% ECIs were positive; +++, 51–75% ECIs were positive; ++++, >75% ECIs were positive.

### Distribution of peroxisome markers at the periphery of ECIs

**Figure 4:**
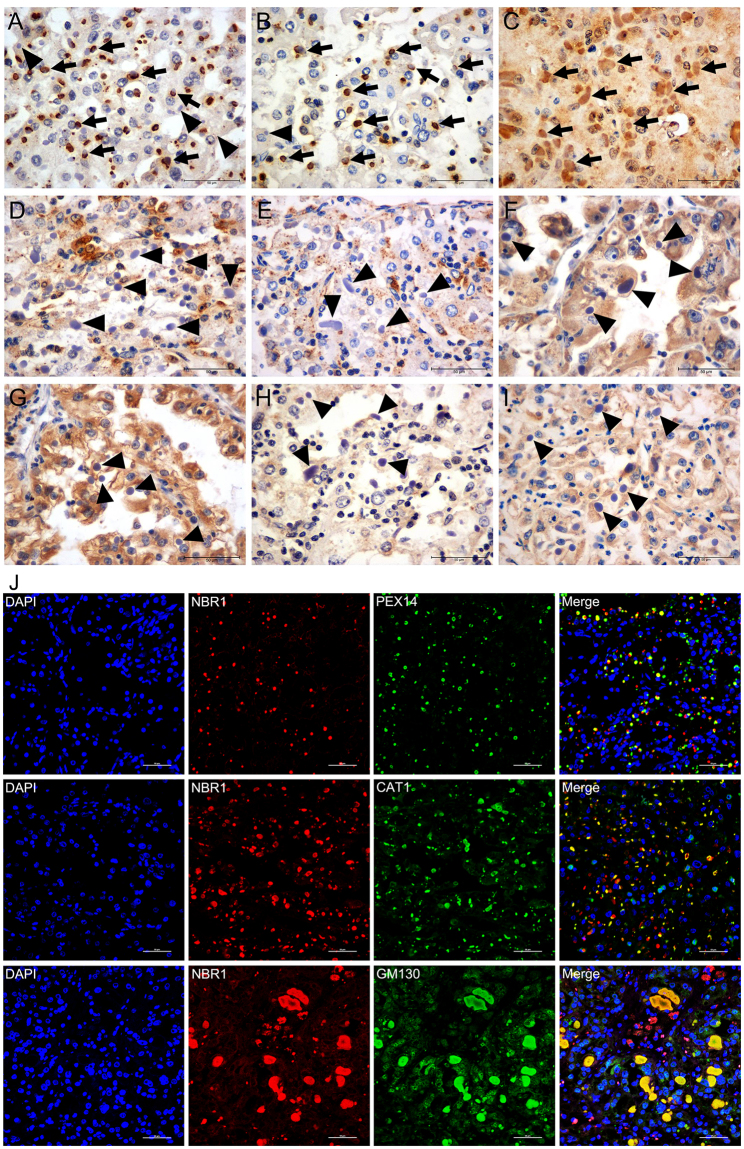
Localization of organelle markers in ECIs of RCCs. Immunohistochemical staining showed that ECIs were intensively positive for PEX14 and CAT1 (two markers of peroxisomes, arrows in **A** and **B**). Some of the ECIs were positive for GM130 (a marker of Golgi apparatus, arrows in **C**). None of the ECIs exhibited immunoreactivity of LAMP1 and LAMP2 (two markers of lysosome or autolysosome, arrowheads in **D** and **E**), TOM20 (a marker of mitochondria, arrowheads in **F**), CALR (a marker of the endoplasmic reticulum, arrowheads in **G**), RPS6 (a marker of ribosomes, arrowheads in **H**), or RAB5A (a marker of early endosomes, arrowheads in **I**). Double- immunofluorescence labelling of ECIs revealed that most of the NBR1-positive ECIs were positive for PEX14 and CAT1, and that a portion of them displayed moderate GM130 staining (**J**).

To determine the nature of the single membrane-bound electron-dense structures located at the periphery of ECIs, samples with (patient 6, 7, 8, and 9) and without (patient 4 and 5) the electron-dense structures were chosen and labeled with antibodies specific to different cell organelles. The remaining four samples (patient 1, 2, 3, and 10) not underwent ultrastructural analysis were subjected to immunostaining for the organelles as well. Immunoreactivity for PEX14 and CAT1 (markers of peroxisomes) were observed in the majority of ECIs, which usually displayed a ring-like or a semi-circle-shaped reactive periphery with a less intense or non-staining center (patient 6, 7, 8, and 9; Fig. 4A,B). Local immunoreactivity could also be visualized in the ECIs (patient 6, 7, 8, and 9; Fig. 4A,B). Interestingly, the antigenic distribution of PEX14 and CAT1 correlated with the electron-dense organelles with single membrane observed by ultrastructural analysis. Moreover, PEX14 and CAT1 were not detected in ECIs without surrounding electron-dense structures examined by TEM (patient 4 and 5). Surprisingly, GM130 immunoreactivity was also observed in ECIs (patient 6, 7, 8, and 9; Fig. 4C) in the form of homogenous staining of the entire inclusion body. Nevertheless, GM130 antigenic distribution was significantly different from that of PEX14 and CAT1. This indicates that proteins from the Golgi apparatus may also have been sequestered into the inclusions and serve as components of the proteinaceous materials. Moreover, this result indicates that the electron-dense organelles were not Golgi apparatus. In stark contrast, no antigenic expression of LAMP1 (Fig. 4D), LAMP2 (Fig. 4E), TOM20 (Fig. 4F), CALR (Fig. 4G), RPS6 (Fig. 4H), or RAB5A (Fig. 4I) was detected in ECIs. The detailed labeling profiles of different organelle markers in ECIs are shown in Table 2. Double-immunofluorescence staining showed that NBR1 was partially or totally co-localized with PEX14 or CAT1 (Fig. 4J). Further, PEX14 and CAT1 was not detected in NBR1-negative ECIs (Table 2). The GM130 protein and NBR1 also co-existed in ECIs (Fig. 4J). These results indicate that the single membrane-bound electron-dense structures within the ECIs were actually peroxisomes, and that NBR1 present in ECIs mainly played a role in the recruitment of peroxisomes to the inclusion bodies.

### Three-dimensional visualization of ECIs

**Figure 5:**
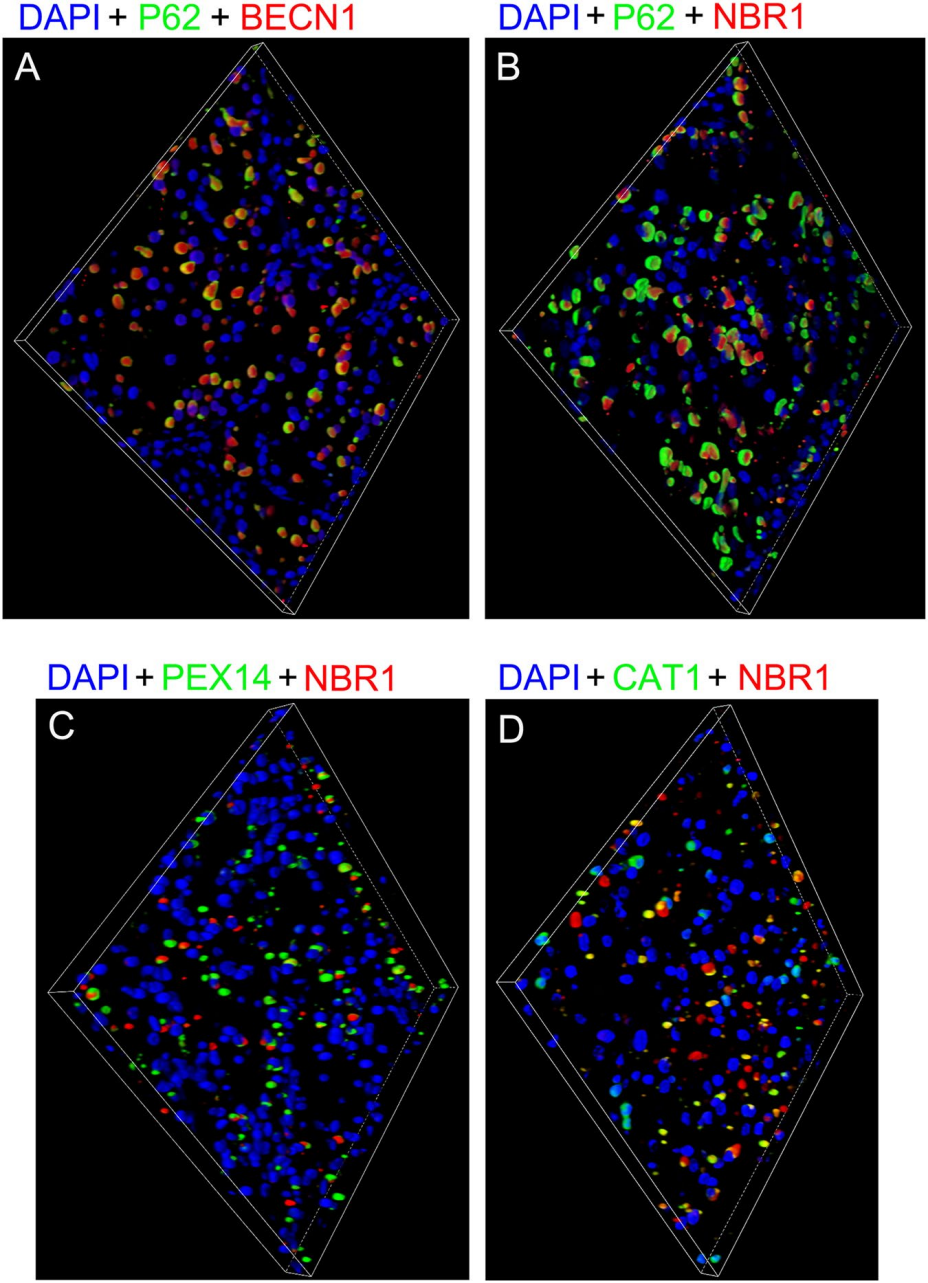
Three-dimensional reconstruction images of ECIs. (**A**) ECIs without NBR1 and peroxisomes. (**A**–**D**) ECIs comprising P62, BECN1, and NBR1 with peroxisomes partially or completely surrounding them.

To obtain sharper and clearer images, thin sections (5–7 μm thickness) were used for three-dimensional rendering and reconstruction. All the images shown here were rotated at a certain angle to facilitate observation. ECIs were composed of P62, BECN1, NBR1, peroxisomes and some other proteins (Fig. 5A–D). They appeared as round to oval bodies (Fig. 5A). BECN1 and NBR1, which represented fibrillar proteins, were located in the central part of ECIs, while P62 was mainly distributed at the margins of ECIs (Fig. 5A,B). Peroxisomes (indicated by PEX14 and CAT1) were associated with NBR1 and partially or completely surrounded ECIs. ECIs without NBR1 and peroxisomes are shown in Fig. 5A. Unfortunately, we were unable to reconstruct the ECIs’ concentric membranous structures because of technical limitations.

### Somatic mutations in ATG genes

All the coding regions of *ATG5*, *ATG7*, *BECN1*, and KEAP1 were amplified for Sanger sequencing. One heterozygous somatic mutation (c.493 T > C in NM_004849.3) was identified in *ATG5* in patient 2, which results in a Pfe-to-Leu substitution at amino acid position 165, a highly-conserved residue in the ATG5 protein. Moreover, a new missense mutation (c.1356 G > T, p.E452D) was also observed in the *ATG7* gene (patient 5 and patient 8). None of the above somatic mutations was detected in the 103 renal tumour samples without ECIs. The results are summarized in Table 3 and the representative Sanger sequencing chromatograms are shown in Supplementary Fig. 3. Besides, we analyzed 1035 renal tumours (including 742 ccRCCs and 293 PRCCs) without ECIs obtained from The Cancer Genome Atlas (TCGA) via the cBioPortal website (www.cbioportal.org). No somatic mutation was found in *ATG5*. The frequency of somatic mutations in *ATG7* was 0.7%. But the nucleotide changes found in renal tumour tissues from TCGA database were not identical to the one observed in this study. No missense nucleotide change was identified in *BECN1* and *KEAP1* (data not shown).

**Table 3:** Validated somatic mutations in ATG genes in ECI-containing DNA samples.

Patient	Gene	Genomic alteration (GRCg37/hg19)	Transcript	Nucleotide change (cDNA)	Amino acid change	Germline result	103 RCCs without ECIs results	1035 RCCs without ECIs in TCGA database
2	ATG5	g.chr6:106958511 T > C	NM_004849.3	c.493 T > C	p.F165L	Absent in normal	Absent	Absent
5	ATG7	g.chr3:113495941 G > T	NM_006395.2	c.1356 G > T	p.E452D	Absent in normal	Absent	Absent
8	ATG7	g.chr3:113495941 G > T	NM_006395.2	c.1356 G > T	p.E452D	No matched normal	Absent	Absent

### Analysis of SNPs in ATG genes

Exome data were analyzed in two patients (patient 6 and patient 7). Of the total mappable reads, 57% were on-target reads, and of the total target bases, 97% were covered at a 20× read depth. Following a series of quality-control steps, 9,367 SNPs that could cause amino acid changes were identified in the coding regions of patient 7. Of these SNPs, 232 were novel. While in patient 6, 185 novel SNPs that could lead to amino acid substitutions were detected. Unfortunately, none of the genes containing novel SNPs was ATG gene. No somatic mutations were identified in ATG genes in these two patients. Of note, four known SNPs in *ATG10*, *RB1CC1*, and *ATG16L1* were detected. And they were chosen for further validation in all ten tumour samples and matched normal renal tissues by Sanger sequencing. The results are summarized in Supplementary Table 2 and the representative Sanger sequencing chromatograms of validated nucleotide changes are shown in Supplementary Fig. 3. Two genetic variants of *ATG10* (rs1864182 [p.P220H] and rs1864183 [p.T212M]) were observed in all ten patients. Moreover, 70% of the variations were homozygous. The variant rs17337252 (p.M234T) of *RB1CC1* was detected in 9 patients, only two of whom were heterozygous for the variant. The *ATG16L1* SNP rs2241880, which encodes a missense variant that leads to a threonine-to-alanine change at residue 300 (p.T300A), was detected in four patients, two of whom were homozygous for the substitution.

## Discussion

To the best of our knowledge, the impact of autophagy defects on human RCC has not been reported so far. In the present study, which we believe is the first of its kind, we report that ECIs found in RCCs were aggregates of proteinaceous material and/or peroxisomes which represents a secondary change upon established RCC subtypes based on the findings of ultrastructural analysis and immunostaining. The proteinaceous material comprised P62 along with a few other ATG proteins. These results indicate that ECIs observed in RCCs are the consequence of autophagy defects. In addition, clustered peroxisomes were found distributed at the periphery of ECIs. While peroxisomes are mainly degraded through pexophagy^25–27^, accumulation of peroxisomes further confirmed the involvement of an autophagy defect^28,29^ in RCC. Even though we have not been able to demonstrate the exact effects of ECIs and autophagy defect on RCCs, we have for the first time directly linked autophagy defect to the characteristic morphologic features of microscopically visible ECIs in human RCC.

The precise mechanism of inclusion body formation is rather complex and largely unknown^30–32^. In the present study, P62 was detected in the majority of ECIs. This may indicate that P62 plays a critical role in ECI formation. An *in vitro* study had proven that transfection of *P62* could lead to fibrillar aggregate formation^33^. Moreover, no protein aggregates formed in autophagy-deficient cells when *P62* was knocked out^34^. Besides P62, Ub1 was found to be present in a minority of ECIs, which may suggest that Ub1 is a co-factor in ECI formation. Apart from P62 and Ub1, LC3 was also found in a small proportion of ECIs. LC3 is known to interact with P62. However, the presence of LC3 might not be associated with ECI formation, because knockdown of LC3 expression did not have an impact on inclusion formation^35^. In contrast to LC3 and Ub1, BECN1 and ATG5 were found to be deposited in the majority of ECIs. Interestingly, BECN1 also has the ability to oligomerize^36^, through which small aggregates could be assembled into larger ones. The recruitment of BECN1 and ATG5 to the protein aggregates further increased the volume of aggregates and resulted in ECI formation. Based on the above results, we hypothesized that the aggregation of the identified proteins, except for perhaps LC3, may lead to the formation of ECIs without the presence of the surrounding peroxisomes.

Under certain conditions, NBR1 is also recruited to the inclusions through interactions with P62 or LC3, but this is accompanied by the generation of numerous clustered peroxisomes^23,35,37^. NBR1, which acts as a pexophagy receptor^23,35,37^, probably recruits peroxisomes to the aggregates, and this results in the formation of ECIs with surrounding peroxisomes. In line with the hypothesis above, in the present study, peroxisomes were only detected in NBR1-positive ECIs, and the proportion of NBR1-positive ECIs was identical to the proportion of PEX14-positive ECIs. The results therefore confirm that NBR1 mainly participates in the recruitment of peroxisomes to ECIs. Nevertheless, the specific mechanisms of ECI formation deserve further investigation.

During the process of autophagy, the ATG12-ATG5-ATG16L1 complex performs vital functions in autophagosomal membrane expansion^38–41^. Previous studies have confirmed that loss of *Atg5* in mouse liver leads to the formation of cytoplasmic inclusion bodies and the development of hepatocellular adenomas^34,42,43^. In the present study, one somatic mutation was identified in the *ATG5* gene, which generated a novel protein with a Pfe-to-Leu change at residue 165. And this nucleotide change was not detected in matched normal renal tissues and 103 RCCs without ECIs. We think that this amino acid substitution might result in ATG5 dysfunction, which is associated with a phenotype similar to that of *Atg5* knockout in mice, since the affected codon is highly conserved in a variety of species.

ATG7 serves as an E1-like protein, which mainly mediate the conjugation of ATG5 to ATG12^40,41^ to facilitate ATG12-ATG5-ATG16L1 complex formation. A new somatic mutation (c.1356 G > T [p.E452D]) was detected in *ATG7* gene in two of the ten RCCs with ECIs. While cytoplasmic inclusions are often present in *Atg7*^*−/−*^ mouse neurons^34^, and knockout of *Atg7* in mice causes pexophagy defect^29^. Based on these findings, we think that the genetic variant of ATG7 (c.1356 G > T [p.E452D]) might mediate ECI formation by impeding autophagosomal membrane expansion.

In addition to the somatic mutations identified in *ATG5* and *ATG7* genes, some known SNPs were also present in the RCCs with ECIs. The variants of *ATG10* (rs1864182 [p.P220H] and rs1864183 [p.T212M]), RB1CC1 (rs17337252 [p.M234T]), and ATG16L1 (rs2241880 [p.T300A]) were detected in ten, nine and four out of the ten cases respectively. The high frequencies of the aforementioned SNPs indicate that they may contribute to autophagy defects and inclusion formation.

To sum up the above results, we found that different nucleotide changes occurred at high frequency in a variety of ATG genes, which could be sequentially involved in a range of processes, from isolation membrane formation to autophagosome membrane elongation and enclosure. And the nucleotide changes found in RCCs with ECIs in this study were apparently different from that observed in RCCs without ECIs and in TCGA database, especially the somatic mutations found in *ATG5* and *ATG7* genes. As a result, we believe that the aforementioned variants of ATG genes present in RCCs are probably associated with autophagy dysfunction and promote ECI formation.

One limitation to the present study is the limited number of RCCs with ECIs. But the limitation *per se* is hard for us to overcome, because RCCs with ECIs were extremely rare. Accordingly, statistical analysis was not performed and the relationship between ECI and clinicopathological characteristics was not clearly delineated. Further investigations are required to solve the above problems.

In conclusion, the results indicate that the rare ECIs consisting of P62, NBR1, ATG proteins and/or peroxisomes observed in RCCs are the products of autophagy defects. The ECIs were all present in high Fuhrman grade tumours, which harbored a considerable number of ATG gene variations; this indicates that the genetic variations of the ATG genes were correlated with autophagy defects, ECI pathogenesis, and tumour grade.

## Methods

### Sample collection and morphological examination

Ten formalin-fixed, paraffin-embedded human kidney tumour samples were obtained from Xijing Hospital, Xi’an, China (n = 5); Peking University Third Hospital, China (n = 1); Liaocheng Hospital, China (n = 1); Nanjing General Hospital, China (n = 1); Nanjing Drum Tower Hospital, China (n = 1); and Zhejiang Provincial People’s Hospital, China (n = 1). Of the 10 samples, 8 were identified as ccRCC, 1 was identified as PRCC, and 1 was identified as MTSCC by immunohistochemistry with CD10, CA9, P504S, and CK7 antibodies and FISH with the 3p loss, CEP7 trisomy or CEP17 trisomy probes. Then, 4-μm thick sections were cut and used for hematoxylin and eosin staining. Five pathologists (S.P.G., Q.G.Y., Y.G., X.L. and Z.W.) re-reviewed all the 10 cases. The patient’s clinicopathological characteristics were obtained (Table 1). The histopathologic characteristics evaluated included the presence of ECIs and Fuhrman nuclear grade. Besides, 103 renal carcinoma samples (including 83 ccRCCs, 17 PRCCs, and 3 MTSCCs) were also collected for Sanger sequencing validation. All patients in this study provided their written informed consent for the use of their tumour samples. All methods used in this study were performed in accordance with the approved guidelines and regulations of the Fourth Military Medical University. This study was approved by the ethics committee of Xijing Hospital, Fourth Military Medical University.

### Immunohistochemistry

Tumour tissue blocks from each sample were selected for immunohistochemistry. Briefly, slides were deparaffinized in xylene, and rehydrated in a descending alcohol series (100%, 95%, 80%, 70% and 50%) followed by distilled water. Sections were subjected to heat- and pressure-induced antigen retrieval in sodium citrate buffer (pH 6.0) for 3 min. Endogenous peroxidase activity in tumour sections was quenched with 3% hydrogen peroxide (H_2_O_2_) in methanol for 10 min. Then, the sections were incubated overnight at 4 °C with the following indicated dilutions of antibodies: mouse anti-P62 (1:500; Abcam, ab56416), rabbit anti-NBR1 (1:300; Proteintech, 16004-1-AP), mouse anti-NBR1 (1:50; Abcam, ab55474), rabbit anti-Ub1 (1:200; Abcam, ab7780), mouse anti-LCK (prediluted; Maxim Biotechnologies, MAB-0051), rabbit anti-LC3 (1:100; Proteintech, 4600-1-AP), rabbit anti-BECN1 (1:200; Proteintech, 11306-1-AP), rabbit anti-ATG5 (1:200; Proteintech, 10181-2-AP), rabbit anti-CAT1 (1:600; Abcam, ab16731), rabbit anti-PEX14 (1:400; Proteintech, 10594-1-AP), rabbit anti-GM130 (1:125; Proteintech, 11308-1-AP), rabbit anti-LAMP1 (1:700; Proteintech, 21997-1-AP), mouse anti-LAMP2 (1:600; Santa Cruz Biotechnology, Sc-18822), rabbit anti-TOM20 (1:300; Proteintech, 11802-1-AP), rabbit anti-RPS6 (1:200; Abcam, ab70227), rabbit anti-CALR (1:200; Proteintech, 10292-1-AP), and rabbit anti-RAB5A (1:300; Proteintech, 11947-1-AP). Bound antibodies were localized using the Dako REAL^TM^ EnVision^TM^ kit (Dako). Diaminobenzidine was used as the chromogen. Positive and negative controls were also used. Brightfield images were obtained using a Nikon Eclipse 50i microscope, with adjustments for brightness and contrast made with Photoshop CC (Adobe Systems, San Jose, CA). Staining was graded as follows: negative (0% antibody-positive inclusions), + (1% to 25% antibody-positive inclusions), ++ (26% to 50% antibody-positive inclusions), +++ (51% to 75% antibody-positive inclusions), and ++++ (76% to 100% antibody-positive inclusions).

### Double-immunofluorescence labeling

To explore the nature and components of ECIs, double-immunofluorescence assay was performed on the samples. Sections were initially treated as described for the immunohistochemical analysis. Non-specific antibody-binding interactions were blocked in phosphate-buffered saline (PBS) containing 5% bovine serum albumin (Sigma-Aldrich, A9647) and 0.1% Tween-20 (Sigma-Aldrich, 274348) for 1 h. Sections were then incubated with mixtures of two primary antibodies (P62 and NBR1, P62 and LC3, P62 and BECN1, P62 and ATG5, NBR1 and PEX14, NBR1 and CAT1, or NBR1 and GM130) at 4 °C overnight, at the following dilutions: mouse anti-P62 (1:150; Abcam, ab56416), rabbit anti-NBR1 (1:250; Proteintech, 16004-1-AP), mouse anti-NBR1 (1:40; Abcam, ab55474), rabbit anti-LC3 (1:25; Proteintech, 14600-1-AP), rabbit anti-BECN1 (1:25; Proteintech, 11306-1-AP), rabbit anti-ATG5 (1:30; Proteintech, 10181-2-AP), rabbit anti-CAT1 (1:50; Abcam, ab16731), rabbit anti-PEX14 (1:100; Proteintech, 10594-1-AP), and rabbit anti-GM130 (1:40; Proteintech, 11308-1-AP). After rinsing, the sections were incubated with mixtures of two secondary antibodies (anti-mouse Alexa Fluor 488 [Invitrogen, A21202] and Alexa Fluor 568 [Invitrogen, A10037] or anti-rabbit Alexa Fluor 594 [Invitrogen, A11012] and Alexa Fluor 488 [Invitrogen, A11008]) for 2 h at room temperature at a 1:500 dilution. Nuclei were stained with DAPI (1:500; Roche, 10236276001). Immunofluorescence images and z-stacks of confocal images at a higher magnification (×40) were obtained using a Nikon C2 Confocal microscope (Nikon, Tokyo, Japan). Projection images were created from 14 series with a Z-step interval of 0.4 µm. The NIS-Elements AR 4.50.00 software (Nikon, Tokyo, Japan) was employed for three-dimensional reconstruction. All images were subjected to adjustments for brightness and contrast with Photoshop CC.

### Transmission electron microscopy

Transmission electron microscopy analysis was performed in six cases (patient 4, 5, 6, 7, 8, and 9). Briefly, small fragments extracted from formalin-fixed, paraffin-embedded tissue blocks were deparaffinized, rehydrated through graded ethanol solutions, and fixed for 24 h in 4% glutaraldehyde in 0.1 M phosphate buffer. Then, the tissue was fixed with 1% osmium tetroxide and embedded in epon resin. Cross-sections (800-nm thick) were cut, stained with toluidine blue and observed to identify areas with cytoplasmic inclusions. Ultrathin sections (90 nm) were prepared, stained with uranyl acetate and lead citrate, and examined with a Tecnai G2 Spirit BioTwin transmission electron microscope (FEI company, America).

### Genomic DNA extraction

Formalin-fixed paraffin-embedded tumours with ECIs and matched adjacent normal tissues were obtained by macrodissection (shown in Supplementary Fig. 1). Extraction of genomic DNA from the aforementioned tissues was performed using the QIAamp FFPE DNA Tissue kit (Qiagen, Germantown, MD) according to the manufacturer’s instructions. The concentration and purity of isolated DNA were determined using NanoDrop ND-2000c (Thermo Scientific, Wilmington, DE, USA) and the Qubit 2.0 (Life Technologies, UK). DNA integrity was examined by agarose gel electrophoresis. The genomic DNA samples of 103 renal carcinomas (including 83 ccRCCs, 17 PRCCs, and 3 MTSCCs) without ECIs were also prepared for Sanger sequencing.

### Whole-exome sequencing and bioinformatics analysis

Genomic DNA samples obtained from two ECI-containing samples (patient 6 and 7) were used for whole-exome sequencing at the Beijing Genomics Institute (BGI), Shenzhen, China to identify genetic variations. The remaining eight tumour samples containing ECIs were utilized to verify the aforementioned genetic variations by Sanger sequencing. No whole-exome sequencing was performed on germline DNA. Briefly, exome sequence capture was performed with the Agilent SureSelect Human All ExonV5 (50 Mb) kit. DNA samples were randomly sheared using Covaris S2. Most of the fragmented DNA ranged from 200 to 300 bp in length. Then, adapters were ligated to both ends of the fragments and ligation-mediated PCR was performed. The amplicons were purified and hybridized to the exome array for enrichment. Captured libraries were sequenced on the HiSeq 4000 platform to generate raw image files. And paired-end reads were obtained. Standard bioinformatics analysis was employed for data generated at BGI. Clean data was produced by data filtering on raw data. All clean data of each renal tumour with ECIs was aligned to the human reference genome (GRCh37/HG19) using the Burrows-Wheeler Aligner (BWA) software. Single nucleotide polymorphisms (SNPs) were detected using the GATK (v3.3.0) software. Then the hard-filtering method was utilized to obtain high-confident variant calls. The variants were annotated using the SnpEff tool. The duplicated data were removed using Picard tools. Additional annotations at the variant and gene level were obtained using information from the 1000 Genomes Project, SIFT, PolyPhen2, and dbSNP.

### Polymerase chain reaction and Sanger sequencing

Based on the SNPs identified using whole-exome sequencing, genes (*ATG10*, *RB1CC1*, and *ATG16L1*) with functional relevance to autophagy were selected. The SNPs detected in the above genes were analyzed in all the ten tumour samples with ECIs and matched normal renal tissues by Sanger sequencing. Some other genes (including *ATG5*, *ATG7*, *KEAP1*, and *BECN1*) related to phagophore formation, autophagosome expansion and substrate degradation were also chosen for coding region analysis in all the ten renal tumours with ECIs by Sanger sequencing. The matched normal renal tissues were used as germline control to identify the somatic mutations. And 103 renal carcinoma tissues without ECIs were utilized to test the existence of the somatic mutations found in *ATG5* and *ATG7*. Primers flanking the target sites or the coding region were designed. The primer sequences are listed in Supplementary Table 1. Purified PCR fragments were sequenced bidirectionally on an ABI 3730 (Applied Biosystems, Foster City, CA, USA) capillary sequencer using the BigDye Terminator v3.1 cycle sequencing kit.

## Electronic supplementary material

Supplementary information
