# Supplementary material for: Autophagy defects and related genetic variations in renal cell carcinoma with eosinophilic cytoplasmic inclusions
Source: Sci Rep. 2018 Jul 2;8:9972. doi: 10.1038/s41598-018-28369-y (PMC6028630; doi:10.1038/s41598-018-28369-y)
Supplement: Supplementary file 1 — Supplementary information [file 41598_2018_28369_MOESM1_ESM.pdf]

Supplementary information

Autophagy defects and related genetic variations in renal cell carcinoma with eosinophilic cytoplasmic inclusions

Zhou Yu[1,2,*], Jing Ma[1,*], Xia Li[1], Yixiong Liu[1], Mingyang Li[1], Lu Wang[1], Ming Zhao[3], Huiying He[4], Yifen Zhang[5], Qiu Rao[6], Danhui Zhao[1], Yingmei Wang[1], Linni Fan[1], Peifeng Li[1], Yang Liu[1], Fang Liu[1], Feng Zhang[1], Jing Ye[1], Qingguo Yan[1], Shuangping Guo[1] & Zhe Wang[1]

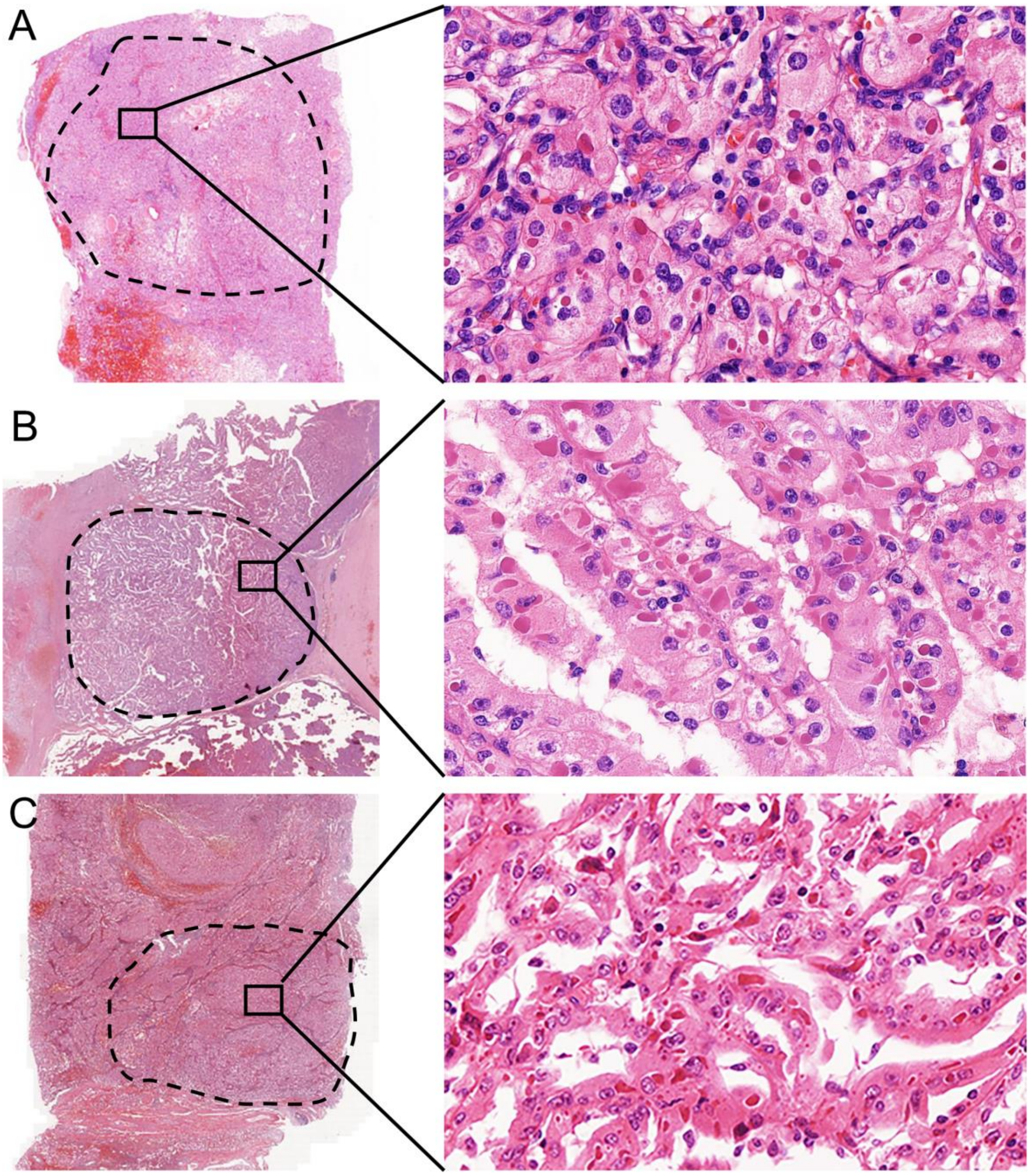

Supplementary Figure 1. Macrodissection of tumour tissues with ECIs. (A) Case1, ccRCC; (B) Case9, PRCC; (C) Case10, MTSCC.

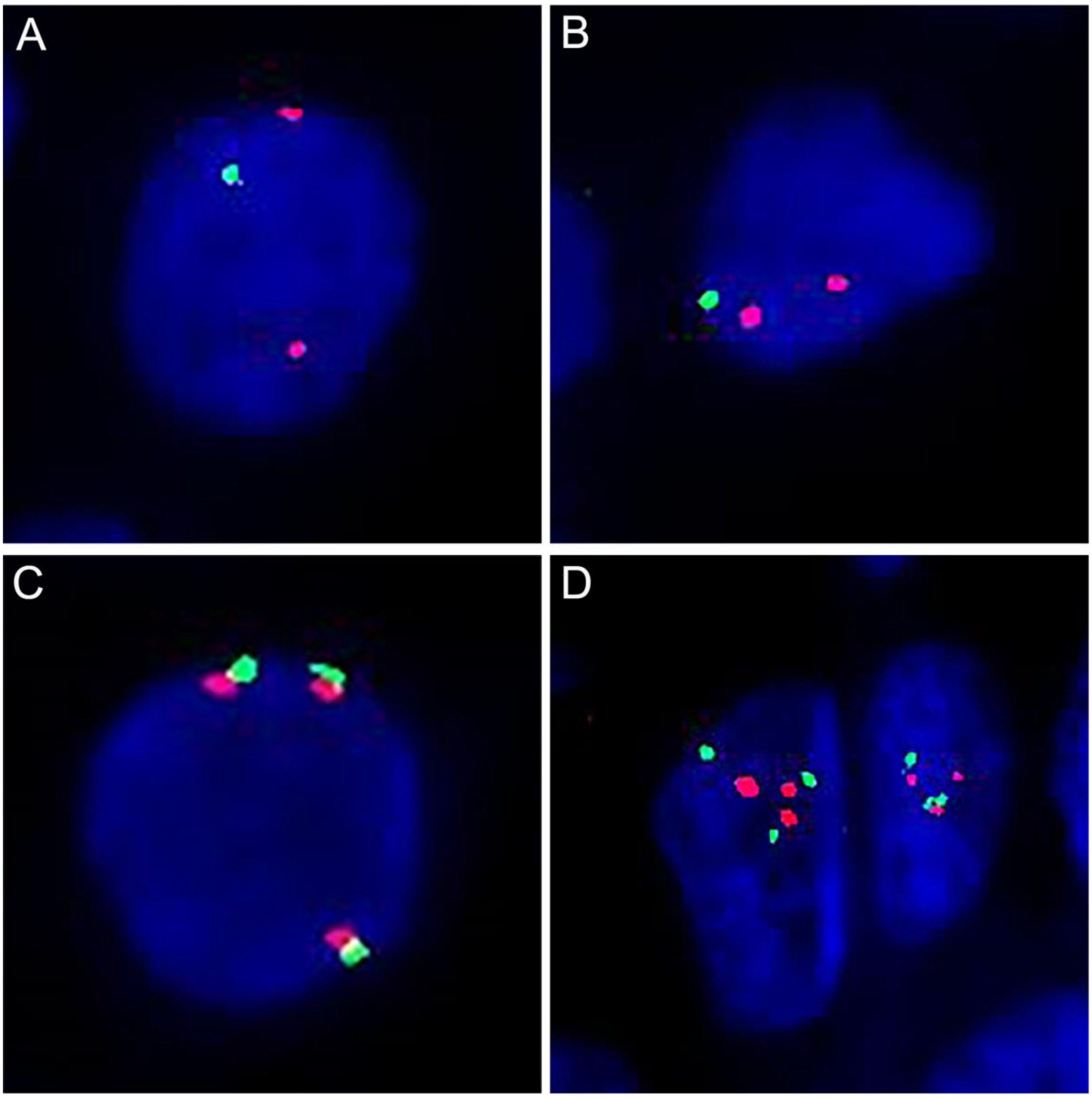

Supplementary Figure 2. Chromosome abnormalities of RCCs. (A, B) Case1 and case 7 showed deletion of chromosome 3p; (C) Case9 displayed trisomy of chromosome 7; (D) Case10 displayed trisomy of chromosome 17.

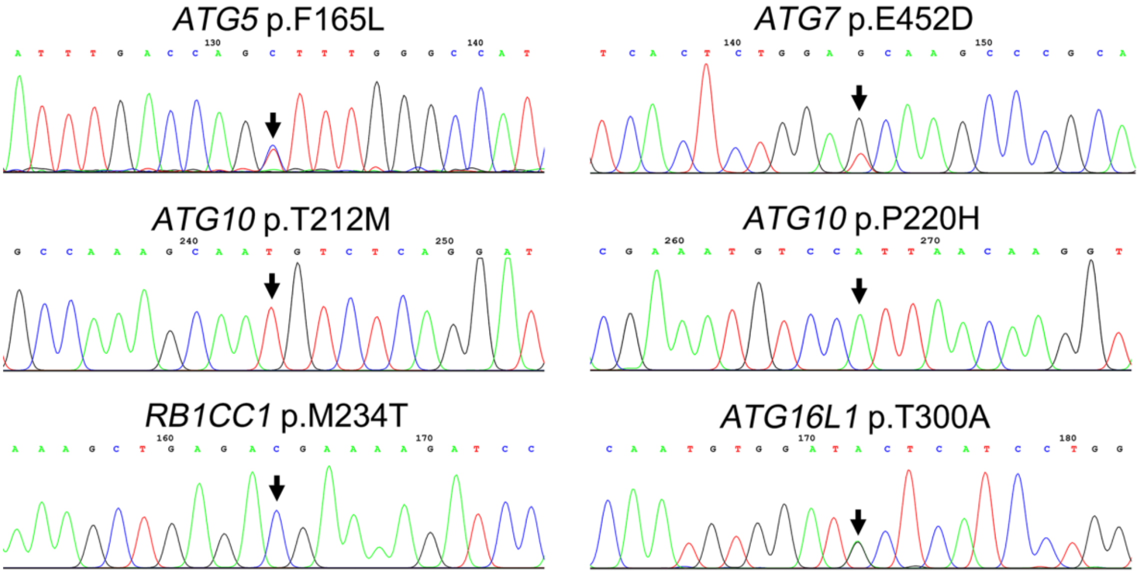

Supplementary Figure 3. Representative Sanger sequencing chromatograms of validated nucleotide changes. Arrow indicates the presence of a given nucleotide change.

Gene	AA Change/Exon	Start	End	Forward primer name	Forward primer sequence	Reverse primer name	Reverse primer sequence	Amplicon size (bp)
ATG5	2	106315905	106316228	ATG5-1F	CTGCTTTAACTCCTGGAAGAATG	ATG5-1R	TGGTTTTGTCCTCTGAGTGGTAG	324
ATG5	3	106308253	106308628	ATG5-2F	AGCAGTAGACTTTGTGTGGGATA	ATG5-2R	GACTAATGACTTCTATCCTCGCA	376
ATG5	4	106292994	106293288	ATG5-3F	ACCTGATGCCTGGAAAGTAGAT	ATG5-3R	AAATGGGACGAAGGAGAAATG	295
ATG5	5	106279585	106279952	ATG5-4F	TGGGTTATTTCAGTGCTAAGAGAT	ATG5-4R	CAACTACTCACAGGGTTATGGCT	366
ATG5	6	106248026	106248360	ATG5-5F	ATGTGATGTCCTTTCAGAAACTTC	ATG5-5R	TTTGGAAAACCCCTAATAAATAC	335
ATG5	7	106201869	106202202	ATG5-6F	TAACACCGTATCAAAAGGCACC	ATG5-6R	AAATATCTTCCTGAAATAACCTGTTG	334
ATG5	8	106186540	106186762	ATG5-7F	GCACTAAGGATTCTCAAAAGGCAC	ATG5-7R	TCAATCTGTTGGCTGTGGGA	223
ATG7	2	11298610	11298903	ATG7-1F	TTATTACAAATGTTCTTTCTCACCAG	ATG7-1R	CGTGAGGATAACAGAAGATGATG	294
ATG7	3	11299264	11299477	ATG7-2F	GGGCTCAACAAAGAGAAGAAAACT	ATG7-2R	TGCTTATTCCTTGCCAAAAGTAGTA	214
ATG7	4	11306913	11307170	ATG7-3F	GCCTGACTAACCGTGTTTCTCTTG	ATG7-3R	TTCCCTTCATATGCCAGTTTCTG	258
ATG7	5	11308946	11309110	ATG7-4F	CCTGGTAACCTGCCTTGATGC	ATG7-4R	GCCAGGGGATTTCACCAACC	165
ATG7	6	11313245	11313548	ATG7-5F	GCATTTCACCTTAAGTTAATGGTGC	ATG7-5R	CACCTTTGGAGGATTTCAGTTACC	304
ATG7	7	11315184	11315575	ATG7-6F	TGGAGGCACTTCTATGTTCACTT	ATG7-6R	ATTTTGCAGTGGTCTCTTGTAAC	392
ATG7	8	11331288	11331578	ATG7-7F	TGTGAAGCTGACATGATACTCG	ATG7-7R	TATGACTGATTTATTGATTACCTGG	291
ATG7	9	11332872	11333150	ATG7-8F	ATTCTCTCCAACTGGTTTGCTGA	ATG7-8R	CTGGTTCCGTTTCTGATAAATGAT	279
ATG7	10	11340588	11340777	ATG7-9F	TCTGCTTGTTTTTATTCTTCCCTC	ATG7-9R	TTACAAAAAGCCCGACTGGAG	190
ATG7	11	11342082	11342348	ATG7-10F	ACAAGATTATTGCATAAAGGAGTG	ATG7-10R	ATGAGAGTAAAACAAGAAGAGAAG	267
ATG7	12	11347806	11348094	ATG7-11F	TGAGATTTCAAGAGACAGCACC	ATG7-11R	TCCCAAGACTTAAACATTTATACATA	289
ATG7	13	11358348	11358636	ATG7-12F	GGAAGTGTGGGCTCTGAGATATT	ATG7-12R	ATCATAATTAGAGACAGGGCCTAC	289
ATG7	14	11360560	11360844	ATG7-13F	TTCATTCCTTGAAACCTGCA	ATG7-13R	CTGCCTCAGTGTACAAGTTGGT	285
ATG7	15	11362726	11362994	ATG7-14F	CTTTGTCACAGAGGATTTCATACTT	ATG7-14R	AGACACTGATGAACAACTGCCTAC	269
ATG7	16	11364546	11364812	ATG7-15F	AGCAAGGGGCATTTTAGTTATCC	ATG7-15R	CAGATGGAATGGCAAAGAGACC	267
ATG7	17	11379922	11380106	ATG7-16F	TGTGGTCGTTGTGTGTTTGATG	ATG7-16R	AATGCTGACCCGCTCACTTT	185
ATG7	18	11426668	11426970	ATG7-17F	CTAGATTGCATTATTATCCCCATGT	ATG7-17R	TAAGCATGTCAGTCTTCACTACAGAA	303

ATG7	19	11554876	11555032	ATG7-18F	GGTGGTATCTTGAATCCCGA	ATG7-18R	GGAGAAGTCAGCCCCACAGC	157
KEAP1	2	10499458	10500060	KEAP1-1F	GTGGTGTTGCTTATCTTCTGGAAC	KEAP1-1R	CTCAGCGAAGTTGGCGATGC	603
KEAP1	2	10499670	10499339	KEAP1-1F1	GTGGTGTCCATTGAGGGTATCC	KEAP1-1R1	TCGTCCATCCCTGGTCCTTC	332
KEAP1	3	10492358	10491972	KEAP1-2F	CTGTCAGCGGCAGTGATAAGTT	KEAP1-2R	CAGGGTGAGCTCCTCGAAGA	387
KEAP1	3	10491511	10492020	KEAP1-2F1	CTCCCGCTGCAAGGACTAC	KEAP1-2R1	TCTCAGTGTCTTGGGACTTGC	510
KEAP1	3	10491999	10492182	KEAP1-2F2	CTGCGAGTCCGAGGTCTTCCAC	KEAP1-2R2	AGGTAGTCCTTGCAGCGGGAGT	183
KEAP1	3	10491516	10491766	KEAP1-2F3	GCAGGAACAACTCGCCCGAC	KEAP1-2R3	GTGTCTTGGGACTTGCCAGGAG	251
KEAP1	4	10489518	10489930	KEAP1-3F	AGAGTATCTGGCCCTTAAGTATTCC	KEAP1-3R	AATCCAGGGCTTCTGTGGTTAC	413
KEAP1	5	10489138	10489378	KEAP1-4F	TGCCCTTTAGGCGTCTGCGT	KEAP1-4R	GCAGTCCACAAAAGATGGGCTA	241
KEAP1	6	10486604	10486892	KEAP1-5F	ATCTCTCTCTTTCTGTCCCCTG	KEAP1-5R	ATGATACTCCCCATTGGACTGT	289
BECN1	2	42823702	42824066	BECN1-1F	GCTCTAAACTGCCTTTGCTCA	BECN-1R	CACCTTCCACATTCTTGACCAC	365
BECN1	3	42820746	42820948	BECN1-2F	GGAGGTGAGGGTGGTGATGA	BECN1-2R	AGGATAGGGGAGAGGGCACT	203
BECN1	4	42819378	42819686	BECN1-3F	TAGTTCTGAGGCTGTTTGGGAG	BECN1-3R	CCATACTGGAAGGTGACGGG	309
BECN1	5	42818656	42818948	BECN1-4F	CTGGCATTGTAGTAGGTGGAGG	BECN1-4R	TGATGTCAAAAAGGTCCCCAGT	293
BECN1	6	42818395	42818738	BECN1-5F	TGGGTAATTGAGCAGGAAGCA	BECN1-5R	TCTAAGATCTCCAAACAGCGTCT	344
BECN1	7	42818189	42818486	BECN1-6F	TTACCCAAGAGCCGTACTCCAC	BECN1-6R	GTGTGAGAAGATAGAACAGGGTGAG	298
BECN1	8	42815797	42816095	BECN1-7F	CCAAGCTAACAGCCTTCAATG	BECN1-7R	GGTGAAGATAACCTACAGTCCCTC	299
BECN1	9	42814415	42814748	BECN1-8F	GGGGTGAGAGTGGGAAATGTC	BECN1-8R	CCAGTCTGTGGGCAGCAAG	334
BECN1	10	42813775	42814078	BECN1-9F	GGGTTGTAACTTCCCAATAATCA	BECN1-9R	TCTACAAGACCCCCAAAACTAAA	304
BECN1	11	42811569	42811906	BECN1-10F	CCAGTGGGATACAGACAGAACAT	BECN1-10R	TACTGTTTTGCCTCCATTATTACTG	338
BECN1	12	42810784	42811056	BECN1-11F	CTATCATTCCTCACCAAGTGTTCT	BECN1-11R	CACCCAAGCAAGACCCCACT	273
ATG16L1	T300A/Exon9	233274553	233275043	ATG16L1-F	AGGACAGGCTATCAACAGAGGC	ATG16L1-R	ATTACTTTTCCTTTGCCCCATC	491
RB1CC1	M234T/Exon7	52673949	52674394	RB1CC1-F	ATAATGTTATGGAGAGGTGGTGAGA	RB1CC1-R	CACCATCTTTAGTGTCAATCGTAGT	446

ATG10	T212M,P220R/H;Exon8	82253123	82253683	ATG10-F	GCTCTCTGGACCTTAAATTCTTGT	ATG10-F	GTAATGCTCGCTTGCTTGCC	561

Supplementary Table 1. List of primers used for Sanger sequencing.

Patient	Gene	Genomic alteration (GRCg37/hg19)	Transcript	Nucleotide change	Amino acid change	Validation	Germline result	Type	SNP NO.
1	RB1CC1	g.chr8:53639020T > C	NM_014781.4	c.701T > C	p.M234T	Validated	No matched normal	MISSENSE	rs17337252
1	ATG10	g.chr5:80982397C > T	NM_031482	c.635C > T	p.T212M	Validated	No matched normal	MISSENSE	rs1864183
1	ATG10	g.chr5:80982421C > A	NM_031482	c.659C> A	p.P220H	Validated	No matched normal	MISSENSE	rs1864182
2	RB1CC1	g.chr8:53639020T > C	NM_014781.4	c.701T > C	p.M234T	Validated	Present in normal	MISSENSE	rs17337252
2	ATG10	g.chr5:80982397C > T	NM_031482	c.635C > T	p.T212M	Validated	Present in normal	MISSENSE	rs1864183
2	ATG10	g.chr5:80982421C > A	NM_031482	c.659C> A	p.P220H	Validated	Present in normal	MISSENSE	rs1864182
3	RB1CC1	g.chr8:53639020T > C	NM_014781.4	c.701T > C	p.M234T	Validated	No matched normal	MISSENSE	rs17337252
3	ATG10	g.chr5:80982397C > T	NM_031482	c.635C > T	p.T212M	Validated	No matched normal	MISSENSE	rs1864183
3	ATG10	g.chr5:80982421C > A	NM_031482	c.659C > A	p.P220H	Validated	No matched normal	MISSENSE	rs1864182
4	ATG10	g.chr5:80982397C > T	NM_031482	c.635C > T	p.T212M	Validated	Present in normal	MISSENSE	rs1864183
4	ATG10	g.chr5:80982421C > A	NM_031482	c.659C > A	p.P220H	Validated	Present in normal	MISSENSE	rs1864182
5	ATG16L1	g.chr2:234189449A > G	NM_030803.6	c.898A > G	p.T300A	Validated	Present in normal	MISSENSE	rs2241880
5	RB1CC1	g.chr8:53639020T > C	NM_031482	c.701T > C	p.M234T	Validated	Present in normal	MISSENSE	rs17337252
5	ATG10	g.chr5:80982397C > T	NM_031483	c.635C > T	p.T212M	Validated	Present in normal	MISSENSE	rs1864183
5	ATG10	g.chr5:80982421C > A	NM_031484	c.659C > A	p.P220H	Validated	Present in normal	MISSENSE	rs1864182
6	ATG16L1	g.chr2:234189449A > G	NM_030803.6	c.898A > G	p.T300A	Validated	Present in normal	MISSENSE	rs2241880

6	RB1CC1	g.chr8:53639020T > C	NM_031482	c.701T > C	p.M234T	Validated	Present in normal	MISSENSE	rs17337252
6	ATG10	g.chr5:80982397C > T	NM_031483	c.635C > T	p.T212M	Validated	Present in normal	MISSENSE	rs1864183
6	ATG10	g.chr5:80982421C > A	NM_031484	c.659C > A	p.P220H	Validated	Present in normal	MISSENSE	rs1864182
7	RB1CC1	g.chr8:53639020T > C	NM_031482	c.701T > C	p.M234T	Validated	Present in normal	MISSENSE	rs17337252
7	ATG10	g.chr5:80982397C > T	NM_031483	c.635C > T	p.T212M	Validated	Present in normal	MISSENSE	rs1864183
7	ATG10	g.chr5:80982421C > A	NM_031484	c.659C > A	p.P220H	Validated	Present in normal	MISSENSE	rs1864182
8	RB1CC1	g.chr8:53639020T > C	NM_031482	c.701T > C	p.M234T	Validated	No matched normal	MISSENSE	rs17337252
8	ATG10	g.chr5:80982397C > T	NM_031483	c.635C > T	p.T212M	Validated	No matched normal	MISSENSE	rs1864183
8	ATG10	g.chr5:80982421C > A	NM_031484	c.659C > A	p.P220H	Validated	No matched normal	MISSENSE	rs1864182
9	ATG16L1	g.chr2:234189449A > G	NM_030803.6	c.898 A > G	p.T300A	Validated	No matched normal	MISSENSE	rs2241880
9	RB1CC1	g.chr8:53639020T > C	NM_031482	c.701T > C	p.M234T	Validated	No matched normal	MISSENSE	rs17337252
9	ATG10	g.chr5:80982397C > T	NM_031483	c.635C > T	p.T212M	Validated	No matched normal	MISSENSE	rs1864183
9	ATG10	g.chr5:80982421C > A	NM_031484	c.659C > A	p.P220H	Validated	No matched normal	MISSENSE	rs1864182
10	ATG16L1	g.chr2:234189449A > G	NM_030803.6	c.898A > G	p.T300A	Validated	Present in normal	MISSENSE	rs2241880
10	RB1CC1	g.chr8:53639020T > C	NM_031482	c.701T > C	p.M234T	Validated	Present in normal	MISSENSE	rs17337252
10	ATG10	g.chr5:80982397C > T	NM_031483	c.635C > T	p.T212M	Validated	Present in normal	MISSENSE	rs1864183
10	ATG10	g.chr5:80982421C > A	NM_031484	c.659C > A	p.P220H	Validated	Present in normal	MISSENSE	rs1864182

Supplementary Table 2. Validated SNPs in ATG genes in ECI-containing DNA samples.
